# The role of dietary patterns and erythrocyte membrane fatty acid patterns on mild cognitive impairment

**DOI:** 10.3389/fnut.2022.1005857

**Published:** 2022-11-04

**Authors:** Xuan Wang, Tiantian Li, Huini Ding, Yuru Liu, Xiaoqiang Liu, Kang Yu, Rong Xiao, Yuandi Xi

**Affiliations:** ^1^Beijing Key Laboratory of Environmental Toxicology, School of Public Health, Capital Medical University, Beijing, China; ^2^Fangshan District Center for Disease Control and Prevention, Beijing, China; ^3^Shijiaying Health Service Center, Beijing, China; ^4^Peking Union Medical College Hospital, Beijing, China

**Keywords:** fatty acid, cognitive impairment, dietary pattern, erythrocyte membrane fatty acid profile, sphingomyelin

## Abstract

**Background:**

Dietary fatty acids have been shown to be associated with the development of cognition. However, research on the role of fatty acid intake in dietary patterns and fatty acid patterns (FAPs) in the development of cognitive function is limited. The aim of this study was to explore the correlation between dietary patterns and FAPs and to provide available evidence for preventing mild cognitive impairment (MCI) through these patterns.

**Materials and methods:**

The 973 participants aged between 65 and 85 were recruited from 2020 to 2021 for this multicenter research in Beijing. Neuropsychological tests were used for cognitive evaluation, and data of dietary intake in the past 12 months were collected with semi-quantitative food frequency questionnaire. The erythrocyte membrane fatty acid profile was tested by chromatography and mass spectrometry lipid profiling. Factor analysis was used to derive the main dietary patterns and FAPs. Pearson’s correlation or Spearman’s correlation was used to explore the association between dietary patterns and FAPs. Binary logistic regression was applied to examine the relationship between patterns and cognitive function.

**Results:**

Six dietary patterns and six FAPs were identified, explaining 53.4 and 80.9% of the total variance separately. After adjusting all potential confounders, T3 of the pattern 1 and FAP2 were the independent protect factors for MCI, respectively (OR 0.601, 95% CI [0.395, 0.914]; OR 0.108, 95% CI [0.019, 0.623]). Rich of SM (26:0), SM (24:1), and SM (26:1) is the characteristic of FAP2. A positive correlation was found between component scores of dietary pattern1 and FAP2 (*r* = 0.441, *p* = 0.001). People who adhered to a reasonable intake of animal flesh consumed more various long-chain fatty acids as well.

**Conclusion:**

The erythrocyte membrane metabolites, SM (26:0), SM (24:1), and SM (26:1), might function as early biomarkers for predicting or monitoring of cognitive aging in the elderly. The dietary pattern with recommended animal flesh consumption was significantly associated with FAP characterized by very long-chain SMs. This dietary pattern affected FAP, which might achieve the ultimate goal of neuroprotection through the very long-chain SMs. A rational intake of dietary fatty acids might be an effective way on preventing MCI in the elderly.

## Introduction

Mild cognitive impairment (MCI) is a situation in which individuals show cognitive impairment with minimal damage of instrumental activities of daily living (IADL) (1–3). This refers to an intermediate stage from normal aging to dementia (1). Older adults with MCI have the highest risk of progression to dementia (4). By 2050, it is estimated that there will be 2 billion people aged 60 years and over, and 131 million of whom are expected to be influenced by dementia (5).

Dietary nutrition is an important way to promote healthy aging and prevent age-related diseases (6). In recent years, dietary factors, especially fat intake (7), have been shown to be involved in the development of hippocampal neurogenesis and cognition (8–10). Fatty acids in tissues, the important composition of fat, can reflect both the quantity and quality of dietary fat intake and have been recognized as reliable biomarkers in epidemiologic studies (11). Previous studies have shown associations between intake of dietary nutrients or dietary patterns and cognition function (10, 11). Long-chain polyunsaturated fatty acids (LCPUFAs) have been reported to be potential mediators that might protect nervous system. They also get involved in the mechanisms leading to cognitive impairment or inflammation in elderly subjects (12). However, evidence indicates that saturated fatty acids (SFAs) with different carbon chain lengths have various effects on the process of Aβ generation, and fatty acids with longer chain (C20:0 and C26:0) are more likely to promote Aβ production (13). In addition, dietary fatty acid saturation is reported to be harmful to cognitive function in human studies (14). Studies about the circulating fatty acid patterns (FAPs) further find that neuroprotective potential fatty acids binding to specific phospholipids are more valuable to improve neuro-function (15). For example, the major components of polyunsaturated fatty acids (PUFAs, C20:3, C20:4, C22:5, and C22:6) in sphingomyelin (SM) and ceramide (Cer) are believed to decline more preferentially in the brain of aged mouse. However, compared with the plasma fatty acid profiles, the erythrocyte membrane fatty acid profile could reflect a long-term intake of fatty acids (16–18). Therefore, the aim of this study was to explore dietary patterns with suitable fatty acid intake and FAPs by principal components analysis (PCA), find the correlation of both two patterns, and provide available evidence for preventing MCI.

## Materials and methods

### Study design and participants

Participants aged 65–85 in this study were collected in several centers of our research in Beijing from 2020 to 2021 (ChiCTR2100054969). The workflow and standards were referenced from our prior study (19, 20). Finally, 973 participants were collected in this study, and 50 of them were selected for lipid analyses. This study was carried out in accordance with the Declaration of Helsinki and ethically approved by the Ethics Committee of Capital Medical University (Z2019SY052). All informed consents were signed by participants before they were included.

### Cognitive assessment

Cognitive impairment was assessed by the Montreal Cognitive Assessment (MoCA), while mini-mental state examination (MMSE) score was applied to exclude any AD (21). Two-step procedure was used to diagnose MCI individuals according to our previous study (20). Briefly, neurologists would perform a secondary examination of participants to determine the clinical diagnosis, if they were suspected of having MCI based on their MoCA presentation.

### Dietary assessment

The information of dietary intake was collected by the food frequency questionnaire (FFQ) of 2002 China National Nutrition and Health Survey (CNHS 2002) (22) that asked about habitual intake of food over the past year. The energy and nutrient intake were calculated by using the China Food Composition Database (Version 6) (23). The energy-adjusted amounts of all dietary nutrients were calculated by the residual method (24).

### Lipid analysis

Erythrocytes were prepared for lipidomic detection. About 250 μl of water was added into each 50 μl of erythrocyte lysates. After 30s vortex, the samples were frozen and thawed with liquid nitrogen for three times. The samples were then sonicated for 10 min in the ice-water bath. Then, 50 μl of normalized protein concentration of the sample was mixed with 150 μl water and 480 μl extraction liquid (VMTBE: Vmethanol = 5:1) containing internal standard. After 60s vortex, the samples were sonicated for 10 min in the ice-water bath. Then, the samples were centrifuged at 3,000 rpm for 15 min at 4°C. About 250 μl of the supernatant was transferred to a fresh tube. The rest of the sample was added with 250 μl of MTBE, followed by vortex, sonication, and centrifugation, and another 250 μl of the supernatant was taken out. This step was repeated twice. The final supernatants were combined and dried in a vacuum concentrator at 37°C. Then, the dried samples were reconstituted in 100 μl of resuspension buffer (Vdichloromethane: Vmethanol: Vwater = 60:30:4.5) by 30s vortex and sonication on ice for 10 min. The constitution was then centrifuged at 12,000 rpm for 15 min at 4°C, and 30 μl of the supernatant was transferred to a fresh glass vial for LC-MS analysis. The quality control (QC) sample was prepared by mixing 15 μl of the supernatants from all samples.

The UHPLC separation was carried out using a SCIEX ExionLC series UHPLC System. Lipid profiling was performed by a UHPLC system (1290 series, Agilent Technologies, USA) equipped with a Kinetex C18 column (2.1 × 100 mm, 1.7 μm, Phenomen) coupled to Q Exactive (QE)-MS/MS (Thermo Fisher Scientific, Bremen, Germany). Therefore, a binary solvent system consisting of 40% water, and 60% acetonitrile (solvent A) and 10% acetonitrile, and 90% isopropanol (solvent B), both acidified with ammonium acetate (10 mM), was used to establish a gradient elution program following the set of solvent B: 0–12.0 min, 40–100%; 12.0–13.5 min, 100%; 13.5–13.7 min, 100–40%; 13.7–18.0 min, 40%. The injection volume was 4 μl for positive ion mode and 6 μl for negative ion mode, respectively. The column temperature was 40°C. The auto-sampler temperature was 6°C, and the injection volume was 2 μl. Typical ion source parameters were as follows: Ionspray voltage: +5,500/−4,500 V, curtain gas: 40 psi, temperature: 350°C, ion source gas 1:50 psi, ion source gas 2:50 psi, DP: ± 80 V.

In this study, the UHPLC separation was carried out by using a SCIEX ExionLC series UHPLC System. AB Sciex QTrap 6,500 + mass spectrometer was applied for analytical development. Multiple reaction monitoring (MRM) mode was used in mass spectrometry analysis. These analyses resulted in 350 lipids, including 12 SM species and 15 Cer species.

### Statistical analysis

Data of continuous variables were presented as means ± standard deviation (SD) or medians (interquartile ranges, IQR). Discrete variables were expressed as percentages (%). Analysis of variance (ANOVA) or the Kruskal–Wallis rank test was applied for continuous variables, while the chi-square test and Wilcoxon rank-sum test were used for descriptive analysis. Component scores were obtained by dietary pattern of each subject.

PCA was used to identify major dietary patterns and FAPs. The factors were rotated by an orthogonal rotation (varimax) for increasing the explanation and simplifying the structure (25). In the final analysis, factor scores of each participant were produced by multiple regression for each component, and factor loadings were based on the dietary intake or levels of fatty acids.

Factor analysis revealed six major dietary patterns which explained 53.3% of the total variance together in dietary intake. An eigenvalue cutoff > 1, scree plot, and component interpretability were used to decide the number of components to retain. A significant chi-square (*p* < 0.001) for the Bartlett’s test of sphericity and the Kaiser–Meyer–Olkin test > 0.6 could indicate the strong correlation among the variables to allow for factor analysis. Dimension reduction was performed on the original 12 SMs and 15 Cers by PCA. Factor analysis revealed six major FAPs which together explained 80.9% of the total variance.

Tertiles were classified based on the distribution of scores for each pattern across the whole population. They were used to characterize each pattern, build regression models, and so on (26). The effect of each factor on MCI was analyzed by logistic regression model.

For all analyses, the lowest tertile of dietary pattern score or lipid pattern score was considered as reference. Pearson’s correlation and Spearman’s correlation were used to analyze the association between dietary pattern parameters and lipid pattern parameters. Statistical significance was set at a two-sided *p* < 0.05. All statistical analyses were performed through the IBM SPSS Statistics 26. Graphs were drawn using the software program GraphPad Prism 8 and R studio.

## Results

### Demographic characteristics of participants

The demographic characteristics of all subjects are described in Table 1. About 59.8% of 973 participants were female. No difference was found between MCI and control individuals in age and basal metabolic rate (BMR). Compared to control individuals, MCI participants were more likely to be male (*p* = 0.001) and had higher levels of education (*p* < 0.001).

**TABLE 1:** Demographic characteristics of subjects.

	Total	Control	MCI	*p*
*N*	973	442	531	
Age	69 (67, 73)	70 (67, 73)	69 (67, 73)	0.857
Female, *n* (%)	627 (59.8%)	313 (65.1%)	314 (55.4%)	< 0.003**
MoCA score	21 (17, 23)	22 (20, 25)	19 (15, 22)	< 0.001**
BMR, kcal	1,259 (1,156, 1,385)	1,265 (1,157, 1,376)	1,254 (1,155, 1,388)	0.844
Education, *n* (%)				< 0.001**
Illiterate	218 (22.4%)	153 (34.6%)	65 (12.2%)	
Primary school	323 (33.2%)	175 (39.6%)	148 (27.9%)	
Junior high school	350 (36.0%)	81 (18.3%)	269 (50.7%)	
High school and above	82 (8.4%)	33 (7.5%)	49 (9.2%)	

BMR, basal metabolic rate; MCI, mild cognitive impairment. **p < 0.01.

### Dietary pattern

The characteristics of six dietary patterns are shown in Table 2. These were strongly correlated within the pattern, if food groups with absolute factor loading coefficients are greater than or equal to 0.5. Pattern 1, which explained 11.2% of the total variance, was characterized by the consumption of alcohol and animal flesh which included fish, liquor, poultry, and red meat (pork, beef, and mutton). People in pattern 2, which explained 8.8%, were more likely to consume oil, salt, and soy sauce. Pattern 3, which explained 8.7%, had higher consumption of soybean, nuts, vegetables, and coarse grains. Pattern 4, which explained 8.6%, included milk, fruits, and eggs. The characteristic of pattern 5 (explained 8.4%) was that tubers were the main source of potatoes and cereals. Pattern 6, which explained 7.7%, included sugary beverages and desserts.

**TABLE 2:** Factor-loading matrix for the dietary patterns and food groups in sample.

	Pattern 1	Pattern 2	Pattern 3	Pattern 4	Pattern 5	Pattern 6
Fish	0.634*	–0.115	0.175	0.206	–0.086	0.002
Liquor	0.614*	–0.013	–0.201	–0.320	0.165	–0.131
Poultry	0.606*	0.096	–0.067	0.120	0.001	0.003
Red meat	0.600*	–0.013	0.205	0.079	0.139	0.157
Fats and oil	–0.047	0.825*	–0.048	–0.114	0.017	0.046
Condiment	0.049	0.815*	0.09	0.087	0.052	–0.007
Legumes and nuts	0.364	0.001	0.582*	–0.055	–0.098	0.061
Vegetables	0.184	–0.092	0.56*	0.275	0.241	–0.166
Coarse grains	–0.109	0.079	0.546*	0.006	0.071	0.063
Dairy	0.112	–0.03	–0.026	0.677*	–0.213	0.040
Fruits	–0.028	–0.033	0.338	0.590*	0.076	0.027
Eggs	0.287	0.085	–0.358	0.544*	0.230	0.018
Tubers	0.042	0.053	–0.082	–0.078	0.739*	0.122
Wheats and rice	0.056	0.018	0.235	0.021	0.732*	–0.001
Beverages	0.077	–0.050	0.078	–0.025	–0.031	0.790*
Cakes	–0.019	0.087	–0.041	0.088	0.146	0.712*
Percentage of variance explained	11.2%	8.8%	8.7%	8.6%	8.4%	7.7%

*Means factor loading with absolute value ≥0.5.

### The effect of dietary pattern on mild cognitive impairment

The results of the logistic regression analysis are manifested in Table 3. Subjects were divided into three subgroups based on tertiles of factor scores of each dietary pattern. First, six dietary patterns were tested separately after adjusting for age, gender, education, and BMR. Compared with the reference group, T3 of the pattern 1 was an independent protective factor for MCI (OR 0.636, 95% CI [0.443, 0.913]). Besides, T3 of the pattern 3 and T3 of pattern 4 were independent protective factors for MCI (OR 0.678, 95% CI [0.481, 0.956]; OR 0.658, 95% CI [0.467, 0.928]). No difference could be found between MCI and control individuals in other dietary patterns.

**TABLE 3:** Effect of dietary pattern on MCI.

	T1		T2		T3	
			
	OR (95%CI)	*p*	OR (95%CI)	*p*	OR (95%CI)	*p*
Pattern 1	1[Ref.]	NA	0.957 (0.678, 1.352)	0.804	0.636 (0.443, 0.913)	0.014*
Pattern 2	1[Ref.]	NA	0.93 (0.663, 1.305)	0.675	0.884 (0.63, 1.242)	0.478
Pattern 3	1[Ref.]	NA	1.119 (0.794, 1.578)	0.520	0.678 (0.481, 0.956)	0.026*
Pattern 4	1[Ref.]	NA	0.74 (0.526, 1.042)	0.085	0.658 (0.467, 0.928)	0.017*
Pattern 5	1[Ref.]	NA	1.332 (0.948, 1.872)	0.098	1.11 (0.787, 1.565)	0.552
Pattern 6	1[Ref.]	NA	1.082 (0.769, 1.522)	0.652	1.182 (0.839, 1.664)	0.339

Data were all adjusted by age, gender, education, and BMR. OR, odds ratio. *p < 0.05.

### Dietary intake across tertiles of pattern 1, pattern 3, and pattern 4

Compared with T1 participants of pattern 1, the uptake of C14:0, C16:0, C17:0, C18:0, C19:0, C14:1, C15:1, C16:1 (palmitoleic acid, POA), C17:1, and C18:1 (oleic acid, OA) was significantly elevated in T3 of pattern 1 (*p* = 0.001, *p* < 0.001, *p* < 0.001, *p* < 0.001, *p* < 0.001, *p* < 0.001, *p* = 0.005, *p* < 0.001, *p* < 0.001, and *p* = 0.001). They were all long-chain saturated fatty acids (LCSFAs) or long-chain monounsaturated fatty acids (LCMUFAs). However, the consumption of C20:1 and C24:1, which are very long-chain monounsaturated fatty acids (VLCMUFAs), was significantly higher in T1 than T3 of pattern 1 (*p* = 0.004, *p* = 0.003). In addition, most of the LCPUFA and very long-chain polyunsaturated fatty acids (VLCPUFAs), such as C16:2, C20:2, C20:4 (arachidonic acid, AA), C22:3, C22:4, C22:5 (docosapentaenoic acid, DPA), and C22:6 (docosahexenoic acid, DHA), were significantly elevated in T3 of pattern 1 (*p* < 0.001) (Table 4).

**TABLE 4:** Dietary intake across tertiles of dietary pattern 1.

Fatty acids, g/d	Pattern 1		*p*
		
	T1	T3	
Total fatty acid	40.3124 (24.1172, 55.1897)	45.4194 (22.0369, 74.287)	0.014*
SFA	15.1984 (10.4227, 18.0028)	18.1353 (12.7871, 24.2102)	< 0.001**
C14:0	1.1675 (0.7280, 2.2506)	1.3452 (0.6115, 2.4480)	0.001**
C16:0	8.0887 (5.9447, 9.8052)	10.3661 (7.3520, 13.6752)	< 0.001**
C17:0	0.2396 (0.1326, 0.3214)	0.3375 (0.2713, 0.4003)	< 0.001**
C18:0	3.0782 (2.1903, 3.7259)	4.1063 (2.8245, 5.3274)	< 0.001**
C19:0	0.0214 (0.0153, 0.0259)	0.0281 (0.0204, 0.0384)	< 0.001**
MUFA	14.3328 (8.0274, 19.936)	16.1768 (7.6206, 27.999)	0.003**
C14:1	0.0073 (0.0042, 0.0143)	0.0100 (0.0046, 0.0169)	< 0.001**
C15:1	0.0003 (0.0002, 0.0005)	0.0004 (0.0002, 0.0006)	0.005**
C16:1	0.2297 (0.1698, 0.2908)	0.3682 (0.2914, 0.4638)	< 0.001**
C17:1	0.0194 (0.0112, 0.0263)	0.0267 (0.0209, 0.0309)	< 0.001**
C18:1	5.2796 (2.9140, 7.4868)	6.2249 (2.9700, 11.8298)	0.001**
C20:1	0.0694 (0.0000, 0.1534)	0.0071 (0.0000, 0.1800)	0.004**
C24:1	0.0034 (0.0000, 0.0083)	0.0000 (0.0000, 0.0097)	0.003**
PUFA	10.8729 (4.1025, 16.7700)	9.3347 (0.7928, 24.5094)	0.996
C16:2	0.0034 (0.0021, 0.0054)	0.0054 (0.0034, 0.0074)	< 0.001**
C20:2	0.0027 (0.0018, 0.0035)	0.0033 (0.0021, 0.0049)	< 0.001**
C20:3	0.0052 (0.0000, 0.0114)	0.0007 (0.0000, 0.0132)	0.003**
C20:4	0.0052 (0.0031, 0.0068)	0.0065 (0.0041, 0.0100)	< 0.001**
C22:3	0.0000 (0.0000, 0.0000)	0.0000 (0.0000, 0.0001)	< 0.001**
C22:4	0.0003 (0.0002, 0.0004)	0.0005 (0.0003, 0.0008)	< 0.001**
C22:5	0.0000 (0.0000, 0.0001)	0.0001 (0.0000, 0.0003)	< 0.001**
C22:6	0.0023 (0.0022, 0.0024)	0.0026 (0.0023, 0.0031)	< 0.001**

T1, the lowest tertile of dietary patterns; T3, the highest tertile of dietary patterns. SFA, saturated fatty acid; MUFA, monounsaturated fatty acid; PUFA, polyunsaturated fatty acid. *p < 0.05, **p < 0.01.

Besides, food group and SM intake across tertiles of pattern 1 are shown in Table 5. Compared with T1, the consumption of fish, poultry, red meat, liquor, and SM was significantly higher in participants in the top tertile of pattern 1 (*p* < 0.001).

**TABLE 5:** Dietary intake across tertiles of dietary pattern 1.

Dietary intake	Pattern 1		*p*
		
	T1	T3	
Fish, g/d	21.00 (12.25, 35.75)	73.13 (47.06, 109.25)	< 0.001**
Liquor, g/d	0.00 (0.00, 3.50)	10.50 (4.88, 21.00)	< 0.001**
Poultry, g/d	1.50 (0.00, 4.50)	14.00 (7, 24.50)	< 0.001**
Red meat, g/d	0.00 (0.00, 0.00)	1.26 (0.00, 32.53)	< 0.001**
SM, mg/d	2.78 (1.57,3.50)	5.66 (4.59,7.27)	< 0.001**

T1, the lowest tertile of dietary patterns; T3, the highest tertile of dietary patterns. SM, sphingomyelin. **p < 0.01.

We also explored the fatty acid profiles in pattern 3 and pattern 4. In T3 of pattern 3, people tended to consume fewer fatty acids (Supplementary Table 1). Compared with T1 participants of pattern 4, the consumption of total fatty acids, MUFA, and PUFA (Supplementary Table 2) was significantly lower in T3 of pattern 4 (*p* < 0.001). However, the results of the consumption of C14:1, C15:1, C16:1 (POA), C17:1, C16:2, C20:4 (AA), and C22:4 were reversed in pattern 4 (*p* < 0.001).

### Between-group differences in SM and Cer FAPs

**FIGURE 1:**
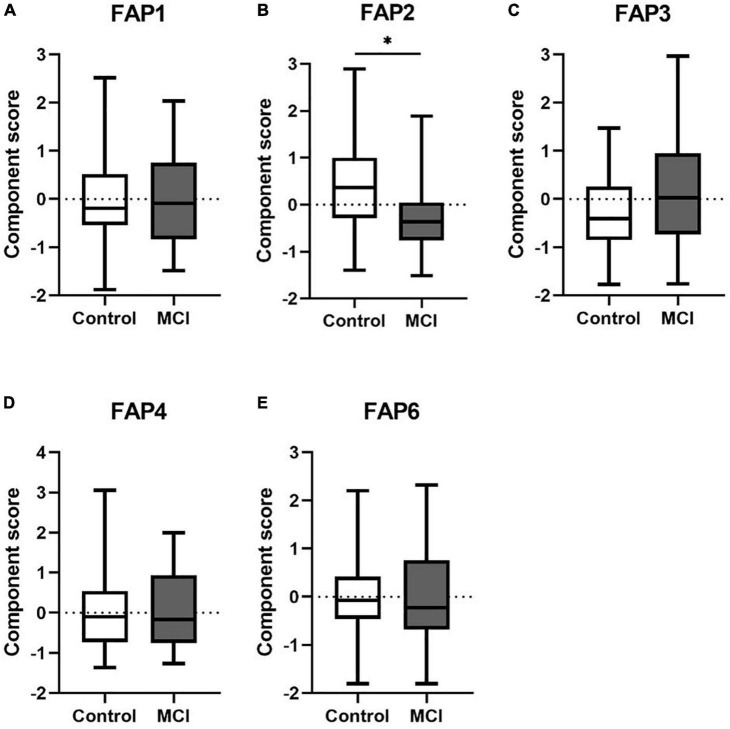
**(A–E)** The differences of FAPs of Cer and SM between control and MCI. Cer, ceramide; SM, sphingomyelin; FAP, fatty acid pattern. *p < 0.05.

Dimension reduction with PCA resulted in six FAPs with eigenvalues > 1 (Supplementary Table 3). The main contributors to each rotational FAP were defined as those with a factor loading > 0.7. All other contributors to each rotational FAP (with a factor loading < 0.7) were excluded from further consideration. Finally, five of six components, FAP1, FAP2, FAP3, FAP4, and FAP6, remained to be further analyzed. Figure 1 illustrates the profile of group difference between control and MCI for FAP2 (*p* < 0.05). FAP2 was characterized by SM (26:0), SM (24:1), and SM (26:1), which were three very long-chain saturated fatty acids (VLCSFAs) and VLCMUFA (Supplementary Table 3).

### Association between dietary patterns or SM intake with FAP2

**FIGURE 2:**
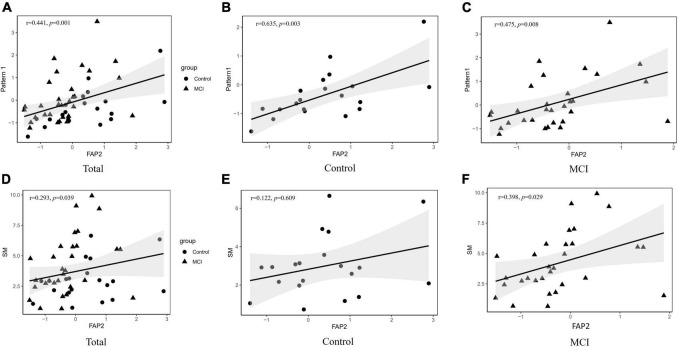
Association between dietary pattern 3 and FAP2. **(A)** The association between component scores of dietary pattern 1 and FAP2 in all subjects. **(B)** The association between component scores of dietary pattern 1 and FAP2 in control. **(C)** The association between component scores of dietary pattern 1 and FAP2 in MCI. **(D)** The association between SM intakes and component scores of FAP2 in all subjects. **(E)** The association between SM intakes and component scores of FAP2 in control. **(F)** The association between SM intakes and component scores of FAP2 in MCI. FAP, fatty acid pattern; SM, sphingomyelin.

Associations between dietary pattern1 or SM intake and FAP2 are shown in Figures 2A–C. The significantly positive correlations were found between component scores of dietary pattern 1 and FAP2 either in total subjects or in each groups, respectively (*r* = 0.441, *p* = 0.001; *r* = 0.635, *p* = 0.003; *r* = 0.475, *p* = 0.008). Furthermore, there was no linear correlation between FAP2 and patterns 3 or 4 (*r* = 0.021, *p* = 0.884; *r* = −0.134, *p* = 0.355). Moreover, the intake of SM was positively correlated with FAP2 overall score (*r* = 0.293 *p* = 0.039) and was also shown in Figure 2, and the same relationship could be found in the consumption of SM with FAP2 scores in MCI (*r* = 0.398, *p* = 0.029).

### The effect of FAP2 on mild cognitive impairment

There were no statistically differences in age, gender, and education among between control and MCI participants who take the quantitative lipidomic analysis (Supplementary Table 4). The results of the logistic regression analysis are manifested in Table 6. Subjects were divided into three subgroups according to the tertiles of FAP2 factor score. Compared with T1 of FAP2, T3 was an independent protective factor for MCI (OR 0.152, 95% CI [0.032, 0.713]). They also showed that the model was improved in predicting MCI when adjusted for BMR. The *p*-value was lower than before, and the odds ratio of T3 on MCI was decreased (adjusted OR 0.108, adjusted 95% CI [0.019,0.623]) as well.

**TABLE 6:** Logistic regression analyses of FAPs for predicting MCI.

	Unadjusted			Adjusted^a^		
		
	β	OR (95%CI)	*p*	β	OR (95%CI)	*p*
FAP1			0.403			0.297
T1	1[Ref.]	1[Ref.]	NA	1[Ref.]	1[Ref.]	NA
T2	−0.724	0.485 (0.122, 1.922)	0.303	−1.100	0.333 (0.072, 1.529)	0.157
T3	0.182	1.200 (0.281, 5.124)	0.806	−0.132	0.876 (0.184, 4.173)	0.868
FAP2			0.024*			0.018*
T1	1[Ref.]	1[Ref.]	NA	1[Ref.]	1[Ref.]	NA
T2	−0.143	0.867 (0.187, 4.007)	0.855	−0.048	0.953 (0.185, 4.907)	0.954
T3	−1.887	0.152 (0.032, 0.713)	0.017*	−2.223	0.108 (0.019, 0.623)	0.013*
FAP3			0.172			0.226
T1	1[Ref.]	1[Ref.]	NA	1[Ref.]	1[Ref.]	NA
T2	−0.608	0.544 (0.137, 2.167)	0.388	−0.465	0.628 (0.147, 2.690)	0.531
T3	0.822	2.275 (0.518, 9.989)	0.276	0.897	2.453 (0.521, 11.558)	0.257
FAP4			0.649			0.571
T1	1[Ref.]	1[Ref.]	NA	1[Ref.]	1[Ref.]	NA
T2	0.671	0.867 (0.187, 4.007)	0.356	0.790	2.204 (0.487, 9.975)	0.305
T3	0.239	0.152 (0.032, 0.713)	0.730	0.180	1.197 (0.285, 5.033)	0.806
FAP6			0.268			0.393
T1	1[Ref.]	1[Ref.]	NA	1[Ref.]	1[Ref.]	NA
T2	−0.857	0.424 (0.104, 1.724)	0.231	−0.819	0.441 (0.102, 1.910)	0.274
T3	0.269	1.309 (0.310, 5.533)	0.714	0.135	1.145 (0.254, 5.169)	0.860

OR, odds ratio; FAP, fatty acid patterns. ^*a*^Data were adjusted by BMR. *p < 0.05.

## Discussion

The relationship between dietary patterns and cognition has received increasing attention due to the complex interactions between various nutrients and food (27–29). The association between circulation or tissue FAPs with cognitive decline has been identified (30, 31). However, few studies have combined dietary patterns with FAPs to explore the association between nutrition and MCI. The main findings of the present study indicated that MCI was cross-sectionally associated with dietary patterns that differ in fatty acid intake. Dietary pattern was closely correlated with erythrocyte membrane FAP. The elderly with higher levels of long-chain SM (LCSMs) had a lower risk of developing MCI.

First, the results showed that males and highly educated people were at higher risk of MCI. This is similar to other studies (32–34). It is indicated that higher education is related with faster cognitive decline on global cognition (35). In this cross-sectional study, six different dietary patterns were identified. Pattern 1 was characterized by high intakes of animal flesh which included fish, liquor, poultry, and red meat (pork, beef, and mutton). It was indicated that people who adhered to a reasonable intake of animal flesh (especially fish) had a lower risk of MCI after considering all potential confounding variables. These foods play a potentially beneficial role in cognition, which have showed in previous studies (36–38). Specifically, we found that participants in the T3 of pattern 1 had much lower ORs on MCI than T1. Moreover, a large longitudinal study demonstrates that lower animal flesh intake is significantly associated with an increased risk of MCI compared with regular intake (39). However, there is another opinion that higher animal flesh consumption leads to high intake of SFA, which can increase inflammation to make a negative impact on cognition (14). In the present study, the intake of poultry plus red meat in the top tertile of pattern 1 score was just up to the recommended amount (300–500 g/week) of the Chinese Dietary Guidelines, and so did fish. It was implied that adequate intake of animal flesh is crucial for neurological health. In addition, less than 40 g/d alcohol consumption has anti-inflammatory effects (40, 41). In general, people in the top tertile of pattern 1 consumed less PUFA, but more total fatty acids, SFA, and MUFA. However, in terms of carbon chain length, those people consumed more LCSFAs, LCMUFAs, and LCPUFAs, but less VLCMUFAs. It showed that VLCMUFAs had no effect on preventing cognitive decline. The percentage of VLCMUFAs and VLCPUFAs in VLCFAs might be related to MCI. Furthermore, an *in vitro* study reports that SFAs with longer chain (C20:0 and C26:0) were more likely to promote Aβ production (42). Therefore, the reasonable ratio of fatty acid intake and its effect on cognitive function for elderly people remains needs more evidence.

The pattern 3 was also positively associated with cognitive functions. The brain-friendly food groups in pattern 3 are legumes, nuts, vegetables, and grains. They are rich in antioxidants such as fiber, beta-carotene, vitamins, folate, and magnesium (38, 43). It has been established that oxidative stress and inflammation contribute to cognitive decline (44). It has been proved that these antioxidants have anti-inflammatory effect and significant association with the prevention of cognitive impairment (45). Our findings also showed that eggs, milk, and dairy products, which are included in pattern 4, are nutritious foods that contain a variety of nutrients associated with improving cognitive function, such as folate, vitamin B12, choline, and protein (38). A systematic review in 2019 indicates that dairy products may help prevent cognitive decline (46). This is consistent with our results. However, fatty acid patterns were no linear correlated with dietary patterns 3 and 4, which might affect cognitive function through the aforementioned non-fatty acid pathways, but it is worth noting that some fatty acids believed having neuroprotection were also found in mode 4 and that might be another way of neuroprotection.

We further focused on LCFAs in our lipidomic results. SM and Cer, as major components of myelin sheath, are related to synaptic dysfunction, neuroinflammation, and neuronal apoptosis in AD (47). Membrane-associated oxidative stress is closely related to activating sphingomyelinases, which cleave SM to generate Cer (48). Excessively, high amounts of ceramide can trigger a form of programmed cell death called apoptosis (48). Cer generated in response to membrane-associated oxidative stress is implicated in the dysfunction and death of cells in a range of disorders, including Alzheimer’s disease and amyotrophic lateral sclerosis (48, 49). Therefore, SMs and Cers were included in PCA in this study. It is certain that reduced plasma concentrations of SM are associated with AD (50, 51). The component score of FAP2 was found significantly different between MCI and control group, and SM (26:0), SM (24:1), and SM (26:1) were the main characteristics of this FAP. Dietary intake of SM was significantly correlated with the levels of SM in FAP2. This result suggested that the levels of SM in the erythrocyte membrane fatty acid profile might be affected by dietary SM intake. A systematic review showed that the results of C26:0 levels are not consistent. One study suggests that aMCI and AD patients have lower levels of C26:0, and another research shows upregulation of C26:0 in erythrocytes in AD subjects (52). One more study demonstrated that the levels of SM (OH) C24:1 and several serum metabolites have significant differences between patients of MCI and early-stage AD and that might be potential biomarkers to distinguish MCI patients who will develop to early-stage AD from stable MCI patients (53). In our linear correlation analysis results, the significantly positive correlations were found between component scores of dietary pattern1 and FAP2 no matter in all subjects or in MCI/control groups, respectively. These results not only demonstrated that erythrocyte membrane fatty acids were affected by long-term dietary habits, but also suggested that dietary fatty acids might exert neuroprotective effects by affecting erythrocyte lipid profiles. This result validated that the dietary patterns with different consumption characteristics of fatty acid might affect FAPs. Our results supported that erythrocyte membrane metabolites, SM (26:0), SM (24:1), and SM (26:1), might function as early biomarkers for predicting or monitoring cognitive aging in elderly (13). Dietary pattern 1 affected FAP2, and this pattern might achieve the ultimate goal of neuroprotection through the very long-chain SMs.

There were some limitations in this study. First, it is the cross-sectional design so that evidence of any causal conclusions between dietary patterns, FAPs, and MCI could not be provided. Therefore, validation in clinical trials was required. Moreover, FFQ is a semi-quantitative questionnaire, and the information of dietary intake depends on subject’s memory, which may lead to recall bias. Finally, it should be carried out with caution to generalize the results we obtained. However, this study combined dietary patterns and FAPs to explore the association between nutrition and MCI. From the perspective of rational intake of dietary fatty acids, the results provided more available data and precise dietary advice to prevent cognitive decline. It provides novel scientific evidence and strategies for nutritional intervention, and it has significant theoretical support and social significance for encouraging healthy aging.

## Conclusion

In conclusion, this study found the suitable dietary patterns and FAPs which have the potential effect on preventing cognitive decline and examined the relationship between dietary patterns and FAPs. It was supported that erythrocyte membrane metabolites, SM (26:0), SM (24:1), and SM (26:1), might function as early biomarkers for predicting or monitoring cognitive aging in the elderly. The dietary pattern with recommended animal flesh intake was significantly associated with FAP characterized by very long-chain SMs. This dietary pattern affected FAP, which might achieve the ultimate goal of neuroprotection through the very long-chain SMs. A rational intake of dietary fatty acids might be an effective way on preventing MCI in the elderly.

## Data availability statement

The raw data supporting the conclusions of this article will be made available by the authors, without undue reservation.

## Ethics statement

The studies involving human participants were reviewed and approved by the Ethics Committee of Capital Medical University. The patients/participants provided their written informed consent to participate in this study.

## Author contributions

XW, TL, and YX contributed to the concept and design of the manuscript. XW and TL performed the statistical analysis. XW was responsible for the drafting of the manuscript. KY, RX, and YX contributed to revision of the manuscript. XW, TL, HD, and YX contributed to the investigation. YL, XL, and YX contributed to project administration. All authors contributed to the article and approved the submitted version.

## References

[1] Petersen RC. Mild cognitive impairment as a diagnostic entity. *J Intern Med.* (2004) 256:183–94. 10.1111/j.1365-2796.2004.01388.x 15324362

[2] Bondi MW Smith GE. Mild cognitive impairment: A concept and diagnostic entity in need of input from neuropsychology. *J Int Neuropsychol Soc.* (2014) 20:129–34. 10.1017/S1355617714000010 24490866 PMC4039178

[3] Petersen RC Lopez O Armstrong MJ Getchius T Ganguli M Gloss D Practice guideline update summary: Mild cognitive impairment: Report of the guideline development, dissemination, and implementation subcommittee of the american academy of neurology. *Neurology.* (2018) 90:126–35. 10.1212/WNL.0000000000004826 29282327 PMC5772157

[4] Salzman T Sarquis-Adamson Y Son S Montero-Odasso M Fraser S. Associations of multidomain interventions with improvements in cognition in mild cognitive impairment: A systematic review and meta-analysis. *JAMA Netw Open.* (2022) 5:e226744. 10.1001/jamanetworkopen.2022.6744 35503222 PMC9066287

[5] Moore K Hughes CF Ward M Hoey L McNulty H. Diet, nutrition and the ageing brain: Current evidence and new directions. *Proc Nutr Soc.* (2018) 77:152–63. 10.1017/S0029665117004177 29316987

[6] Coelho-Junior HJ Trichopoulou A Panza F. Cross-sectional and longitudinal associations between adherence to Mediterranean diet with physical performance and cognitive function in older adults: A systematic review and meta-analysis. *Ageing Res Rev.* (2021) 70:101395. 10.1016/j.arr.2021.101395 34153553

[7] Ding B Xiao R Ma W Zhao L Bi Y Zhang Y. The association between macronutrient intake and cognition in individuals aged under 65 in China: A cross-sectional study. *BMJ Open.* (2018) 8:e18573. 10.1136/bmjopen-2017-018573 29317416 PMC5781185

[8] Murphy T Thuret S. The systemic milieu as a mediator of dietary influence on stem cell function during ageing. *Ageing Res Rev.* (2015) 19:53–64. 10.1016/j.arr.2014.11.004 25481406

[9] de Souza RJ Mente A Maroleanu A Cozma AI Ha V Kishibe T Intake of saturated and trans unsaturated fatty acids and risk of all cause mortality, cardiovascular disease, and type 2 diabetes: Systematic review and meta-analysis of observational studies. *BMJ.* (2015) 351:h3978. 10.1136/bmj.h3978 26268692 PMC4532752

[10] Brown TJ Brainard J Song F Wang X Abdelhamid A Hooper L. Omega-3, omega-6, and total dietary polyunsaturated fat for prevention and treatment of type 2 diabetes mellitus: Systematic review and meta-analysis of randomised controlled trials. *BMJ.* (2019) 366:l4697. 10.1136/bmj.l4697 31434641 PMC6699594

[11] Hodson L Skeaff CM Fielding BA. Fatty acid composition of adipose tissue and blood in humans and its use as a biomarker of dietary intake. *Prog Lipid Res.* (2008) 47:348–80. 10.1016/j.plipres.2008.03.003 18435934

[12] Janssen CI Kiliaan AJ. Long-chain polyunsaturated fatty acids (LCPUFA) from genesis to senescence: The influence of LCPUFA on neural development, aging, and neurodegeneration. *Prog Lipid Res.* (2014) 53:1–17. 10.1016/j.plipres.2013.10.002 24334113

[13] Huo Z Rana BK Elman JA Dong R Engelman CD Johnson SC Metabolic profiling of cognitive aging in midlife. *Front Aging Neurosci.* (2020) 12:555850. 10.3389/fnagi.2020.555850 33250761 PMC7674168

[14] Tan BL Norhaizan ME. Effect of high-fat diets on oxidative stress, cellular inflammatory response and cognitive function. *Nutrients.* (2019) 11:2579. 10.3390/nu11112579 31731503 PMC6893649

[15] Tu J Yin Y Xu M Wang R Zhu ZJ. Absolute quantitative lipidomics reveals lipidome-wide alterations in aging brain. *Metabolomics.* (2017) 14:5. 10.1007/s11306-017-1304-x 30830317

[16] Djousse L Biggs ML Lemaitre RN King IB Song X Ix JH Plasma omega-3 fatty acids and incident diabetes in older adults. *Am J Clin Nutr.* (2011) 94:527–33. 10.3945/ajcn.111.013334 21593500 PMC3142727

[17] Huang NJ Pishesha N Mukherjee J Zhang S Deshycka R Sudaryo V Genetically engineered red cells expressing single domain camelid antibodies confer long-term protection against botulinum neurotoxin. *Nat Commun.* (2017) 8:423. 10.1038/s41467-017-00448-0 28871080 PMC5583347

[18] Fote G Wu J Mapstone M Macciardi F Fiandaca MS Federoff HJ. Plasma sphingomyelins in Late-Onset Alzheimer’s disease. *J Alzheimers Dis.* (2021) 83:1161–71. 10.3233/JAD-200871 34397408 PMC9788856

[19] An Y Zhang X Wang Y Wang Y Liu W Wang T Longitudinal and nonlinear relations of dietary and Serum cholesterol in midlife with cognitive decline: Results from EMCOA study. *Mol Neurodegener.* (2019) 14:51. 10.1186/s13024-019-0353-1 31888696 PMC6937942

[20] Zhang X Wang Y Liu W Wang T Wang L Hao L Diet quality, gut microbiota, and microRNAs associated with mild cognitive impairment in middle-aged and elderly Chinese population. *Am J Clin Nutr.* (2021) 114:429–40. 10.1093/ajcn/nqab078 33871591

[21] Qin H Zhu B Hu C Zhao X. Later-Onset hypertension is associated with higher risk of dementia in mild cognitive impairment. *Front Neurol.* (2020) 11:557977. 10.3389/fneur.2020.557977 33324316 PMC7726443

[22] He Y Ma G Zhai F Li Y Hu Y Feskens EJ Dietary patterns and glucose tolerance abnormalities in Chinese adults. *Diabetes Care.* (2009) 32:1972–6. 10.2337/dc09-0714 19675202 PMC2768212

[23] Ma YH Shen XN Xu W Huang YY Li HQ Tan L A panel of blood lipids associated with cognitive performance, brain atrophy, and Alzheimer’s diagnosis: A longitudinal study of elders without dementia. *Alzheimers Dement (Amst).* (2020) 12:e12041. 10.1002/dad2.12041 32995461 PMC7507431

[24] Willett WC Howe GR Kushi LH. Adjustment for total energy intake in epidemiologic studies. *Am J Clin Nutr.* (1997) 65(4 Suppl):1220S–8S. 10.1093/ajcn/65.4.1220S 9094926

[25] Willey J Wakefield M Silver HJ. Exploring the diets of adults with obesity and type II diabetes from nine diverse countries: Dietary intakes, patterns, and quality. *Nutrients.* (2020) 12:2027. 10.3390/nu12072027 32650448 PMC7400897

[26] Liu D Zhao LY Yu DM Ju LH Zhang J Wang JZ Dietary patterns and association with obesity of children aged 6(-)17 years in medium and small cities in china: findings from the CNHS 2010(-)2012. *Nutrients.* (2018) 11:3. 10.3390/nu11010003 30577428 PMC6356437

[27] Mantzorou M Vadikolias K Pavlidou E Tryfonos C Vasios G Serdari A Mediterranean diet adherence is associated with better cognitive status and less depressive symptoms in a Greek elderly population. *Aging Clin Exp Res.* (2021) 33:1033–40. 10.1007/s40520-020-01608-x 32488472

[28] Trichopoulou A Kyrozis A Rossi M Katsoulis M Trichopoulos D La Vecchia C Mediterranean diet and cognitive decline over time in an elderly Mediterranean population. *Eur J Nutr.* (2015) 54:1311–21. 10.1007/s00394-014-0811-z 25482573

[29] Okubo H Inagaki H Gondo Y Kamide K Ikebe K Masui Y Association between dietary patterns and cognitive function among 70-year-old Japanese elderly: A cross-sectional analysis of the SONIC study. *Nutr J.* (2017) 16:56. 10.1186/s12937-017-0273-2 28893250 PMC5594454

[30] Shen J Li J Hua Y Ding B Zhou C Yu H Association between the erythrocyte membrane fatty acid profile and cognitive function in the overweight and obese population aged from 45 to 75 years old. *Nutrients.* (2022) 14:914. 10.3390/nu14040914 35215564 PMC8878599

[31] Bernath MM Bhattacharyya S Nho K Barupal DK Fiehn O Baillie R Serum triglycerides in Alzheimer disease: Relation to neuroimaging and CSF biomarkers. *Neurology.* (2020) 94:e2088–98. 10.1212/WNL.0000000000009436 32358220 PMC7526673

[32] Fu J Liu Q Du Y Zhu Y Sun C Lin H Age- and Sex-Specific prevalence and modifiable risk factors of mild cognitive impairment among older adults in china: A Population-Based observational study. *Front Aging Neurosci.* (2020) 12:578742. 10.3389/fnagi.2020.578742 33192471 PMC7662098

[33] Qin HY Zhao XD Zhu BG Hu CP. Demographic factors and cognitive function assessments associated with mild cognitive impairment progression for the elderly. *Biomed Res Int.* (2020) 2020:3054373. 10.1155/2020/3054373 32090075 PMC7031731

[34] Yuan Y Li J Zhang N Fu P Jing Z Yu C Body mass index and mild cognitive impairment among rural older adults in China: The moderating roles of gender and age. *BMC Psychiatry.* (2021) 21:54. 10.1186/s12888-021-03059-8 33485307 PMC7825154

[35] van Loenhoud AC Groot C Bocancea DI Barkhof F Teunissen C Scheltens P Association of education and intracranial volume with cognitive trajectories and mortality rates across the Alzheimer disease continuum. *Neurology.* (2022) 98:e1679–91. 10.1212/WNL.0000000000200116 35314498 PMC9052567

[36] Huang Q Jia X Zhang J Huang F Wang H Zhang B Diet-Cognition associations differ in mild cognitive impairment subtypes. *Nutrients.* (2021) 13:1341. 10.3390/nu13041341 33920687 PMC8073801

[37] Zhu J Xiang YB Cai H Li H Gao YT Zheng W A prospective investigation of dietary intake and functional impairments among the elderly. *Am J Epidemiol.* (2018) 187:2372–86. 10.1093/aje/kwy156 30060001 PMC6211247

[38] Huang Q Jiang H Zhang J Jia X Huang F Wang H Dietary patterns are associated with Multi-Dimensional cognitive functions among adults aged 55 and older in china. *Front Nutr.* (2022) 9:806871. 10.3389/fnut.2022.806871 35252296 PMC8891750

[39] Ngabirano L Samieri C Feart C Gabelle A Artero S Duflos C Intake of meat, fish, fruits, and vegetables and Long-Term risk of dementia and alzheimer’s disease. *J Alzheimers Dis.* (2019) 68:711–22. 10.3233/JAD-180919 30883348

[40] Wang X Li T Li H Li D Wang X Zhao A Association of dietary inflammatory potential with blood inflammation: The prospective markers on mild cognitive impairment. *Nutrients.* (2022) 14:2417. 10.3390/nu14122417 35745147 PMC9229190

[41] Imhof A Woodward M Doering A Helbecque N Loewel H Amouyel P Overall alcohol intake, beer, wine, and systemic markers of inflammation in western Europe: Results from three MONICA samples (Augsburg, Glasgow, Lille). *Eur Heart J.* (2004) 25:2092–100. 10.1016/j.ehj.2004.09.032 15571824

[42] Liu JJ Zhang W Wang SS Jia ZQ Shi YH Yang L Effects of chain length of saturated fatty acids on Abeta generation in SH-SY5Y cells. *Neurosci Lett.* (2019) 698:169–72. 10.1016/j.neulet.2019.01.024 30648614

[43] ChanRChanDWooJ. A cross sectional study to examine the association between dietary patterns and cognitive impairment in older Chinese people in Hong Kong. *J Nutr Health Aging.* (2013) 17:757–65. 10.1007/s12603-013-0348-5 24154648

[44] Den H Dong X Chen M Zou Z. Efficacy of probiotics on cognition, and biomarkers of inflammation and oxidative stress in adults with Alzheimer’s disease or mild cognitive impairment – a meta-analysis of randomized controlled trials. *Aging (Albany NY).* (2020) 12:4010–39. 10.18632/aging.102810 32062613 PMC7066922

[45] Jiang X Huang J Song D Deng R Wei J Zhang Z. Increased consumption of fruit and vegetables is related to a reduced risk of cognitive impairment and dementia: Meta-Analysis. *Front Aging Neurosci.* (2017) 9:18. 10.3389/fnagi.2017.00018 28223933 PMC5293796

[46] Cuesta-Triana F Verdejo-Bravo C Fernandez-Perez C Martin-Sanchez FJ. Effect of milk and other dairy products on the risk of frailty, sarcopenia, and cognitive performance decline in the elderly: A systematic review. *Adv Nutr.* (2019) 10:S105–19. 10.1093/advances/nmy105 31089731 PMC6518150

[47] Su H Rustam YH Masters CL Makalic E McLean CA Hill AF Characterization of brain-derived extracellular vesicle lipids in Alzheimer’s disease. *J Extracell Vesicles.* (2021) 10:e12089. 10.1002/jev2.12089 34012516 PMC8111496

[48] Calabrese V Cornelius C Dinkova-Kostova AT Calabrese EJ Mattson MP. Cellular stress responses, the hormesis paradigm, and vitagenes: Novel targets for therapeutic intervention in neurodegenerative disorders. *Antioxid Redox Signal.* (2010) 13:1763–811. 10.1089/ars.2009.3074 20446769 PMC2966482

[49] Perluigi M Di Domenico F Giorgi A Schinina ME Coccia R Cini C Redox proteomics in aging rat brain: Involvement of mitochondrial reduced glutathione status and mitochondrial protein oxidation in the aging process. *J Neurosci Res.* (2010) 88:3498–507. 10.1002/jnr.22500 20936692

[50] Bergland AK Proitsi P Kirsebom BE Soennesyn H Hye A Larsen AI Exploration of plasma lipids in mild cognitive impairment due to Alzheimer’s disease. *J Alzheimers Dis.* (2020) 77:1117–27. 10.3233/JAD-200441 32804144

[51] Oresic M Hyotylainen T Herukka SK Sysi-Aho M Mattila I Seppanan-Laakso T Metabolome in progression to Alzheimer’s disease. *Transl Psychiatry.* (2011) 1:e57. 10.1038/tp.2011.55 22832349 PMC3309497

[52] Cisbani G Bazinet RP. The role of peripheral fatty acids as biomarkers for Alzheimer’s disease and brain inflammation. *Prostaglandins Leukot Essent Fatty Acids.* (2021) 164:102205. 10.1016/j.plefa.2020.102205 33271431

[53] Weng WC Huang WY Tang HY Cheng ML Chen KH. The differences of serum metabolites between patients with Early-Stage Alzheimer’s disease and mild cognitive impairment. *Front Neurol.* (2019) 10:1223. 10.3389/fneur.2019.01223 31824405 PMC6884031

