# Supplementary material for: The role of dietary patterns and erythrocyte membrane fatty acid patterns on mild cognitive impairment
Source: Front Nutr. 2022 Nov 4;9:1005857. doi: 10.3389/fnut.2022.1005857 (PMC9673906; doi:10.3389/fnut.2022.1005857)
Supplement: Supplementary file 1 [file Data_Sheet_1.pdf]

Table S1 Dietary fatty acids intake across tertiles of dietary pattern 3

	Pattern 3		*p*
	T1	T3	
Fatty acids, g/d			
Total fatty acid	50.6113(33.0186, 64.4352)	33.2368(15.7177, 52.1755)	<0.001**
SFA	17.8599(13.9352, 22.8447)	14.7011(9.7764, 18.5665)	<0.001**
C14:0	1.5983(0.8982, 2.5855)	1.0747(0.4881, 1.9915)	<0.001**
C15:0	1.3842(1.2271, 1.5447)	1.1128(0.9274, 1.2811)	<0.001**
C16:0	9.7744(7.6378, 12.3007)	8.0878(5.5889, 10.2644)	<0.001**
C17:0	0.3388(0.2701, 0.393)	0.2517(0.1424, 0.3158)	<0.001**
C18:0	3.7738(2.9976, 4.7938)	3.0567(2.0462, 4.0771)	<0.001**
C19:0	0.0245(0.0191, 0.029)	0.0273(0.018, 0.0401)	<0.001**
C20:0	0.1721(0.0849, 0.2594)	0.0855(0.0000, 0.2002)	<0.001**
C22:0	0.1552(0.0573, 0.2714)	0.0462(0.0000, 0.1963)	<0.001**
MUFA	18.1677(10.8307, 23.8589)	12.0862(4.8204, 19.0414)	<0.001**
C14:1	0.0103(0.0055, 0.0168)	0.0075(0.0026, 0.0132)	<0.001**
C15:1	0.0004(0.0003, 0.0005)	0.0003(0.0002, 0.0005)	<0.001**
C16:1	0.3102(0.2404, 0.3883)	0.2679(0.1804, 0.3467)	<0.001**
C17:1	0.0272(0.0215, 0.0315)	0.0204(0.0122, 0.0259)	<0.001**
C18:1	6.8448(4.1074, 9.1556)	4.4625(1.6702, 7.4016)	<0.001**
C20:1	0.0941(0.0116, 0.1948)	0.0000 (0.0000, 0.093)	<0.001**
C22:1	1.2391(1.0282, 1.4441)	0.9975(0.814, 1.2609)	<0.001**
C24:1	0.0048(0.000, 0.0106)	0.0000 (0.0000, 0.0047)	<0.001**
PUFA	12.4121(6.2923, 19.9357)	6.6768(0.0000, 15.7831)	<0.001**
C16:2	0.0048(0.0032, 0.0065)	0.0038(0.0021, 0.0057)	<0.001**
C18:2	3.8708(0.9136, 7.1575)	1.2578(0.0000, 5.5312)	<0.001**
C18:3	1.1649(0.9583, 1.3712)	1.0687(0.8425, 1.3621)	0.013**
C20:2	0.0036(0.0027, 0.0046)	0.0023(0.0012, 0.0033)	<0.001**
C20:3	0.0071(0.001, 0.0144)	0.0000 (0.0000, 0.007)	<0.001**
C20:4	0.0074(0.0054, 0.0093)	0.0042(0.0021, 0.0062)	<0.001**
C20:5	0.0151(0.0022, 0.0314)	0.0000 (0.0000, 0.0195)	<0.001**
C22:4	0.0004(0.0003, 0.0005)	0.0003(0.0002, 0.0005)	0.001**

T1, the lowest tertiles of dietary patterns; T3, the highest tertiles of dietary patterns. SFA, saturated fatty acid; MUFA, monounsaturated fatty acid; PUFA, polyunsaturated fatty acid. $^{*}p < 0.05$, $^{**}p<0.01$.

Table S2 Dietary fatty acids intake across tertiles of dietary pattern 4

	Pattern 4		*p*
	T1	T3	
Fatty acids, g/d			
Total fatty acid	46.9856(23.9352, 62.8692)	36.2052(23.2574, 52.2562)	<0.001**
SFA	14.6552(9.6151, 17.9221)	18.7942(15.2884, 22.5947)	<0.001**
C14:0	0.846(0.4214, 1.1411)	2.5698(1.8826, 2.9009)	<0.001**
C15:0	1.2138(0.9856, 1.4069)	1.2851(1.1073, 1.4386)	0.006**
C16:0	8.5194(5.6908, 10.3624)	9.833(7.6895, 11.8634)	<0.001**
C17:0	0.1948(0.1239, 0.3116)	0.3256(0.2857, 0.3694)	<0.001**
C18:0	3.2198(2.116, 4.0719)	3.832(3.0061, 4.6155)	<0.001**
C19:0	0.0230(0.0168, 0.0306)	0.0242(0.0179, 0.0318)	0.002**
C20:0	0.1834(0.0652, 0.2618)	0.0851(0.0165, 0.1706)	<0.001**
C22:0	0.1692(0.0294, 0.2909)	0.0307(0.0000, 0.1306)	<0.001**
MUFA	17.1306(8.6487, 23.2287)	12.4904(7.2613, 18.309)	<0.001**
C14:1	0.0058(0.0025, 0.008)	0.0167(0.0118, 0.0192)	<0.001**
C15:1	0.0003(0.0002, 0.0003)	0.0006(0.0004, 0.0007)	<0.001**
C16:1	0.2425(0.1671, 0.3216)	0.3467(0.2859, 0.4243)	<0.001**
C17:1	0.0155(0.0103, 0.0246)	0.0268(0.0232, 0.0301)	<0.001**
C18:1	6.6631(3.3526, 9.1185)	4.5299(2.5033, 6.8502)	<0.001**
C20:1	0.0977(0.0000, 0.203)	0.0000 (0.0000, 0.0600)	<0.001**
C22:1	1.2494(1.0327, 1.475)	0.9627(0.8125, 1.1392)	<0.001**
C24:1	0.005(0.0000, 0.0111)	0.0000 (0.0000, 0.0028)	<0.001**
PUFA	14.2121(5.6546, 20.6304)	4.9921(0.0000, 11.5044)	<0.001**
C16:2	0.0032(0.0019, 0.0047)	0.0063(0.0048, 0.0076)	<0.001**
C18:2	4.6718(1.0545, 7.6314)	0.2514(0.0000, 3.1347)	<0.001**
C18:3	1.2475(1.0098, 1.4619)	0.9848(0.811, 1.1813)	<0.001**
C20:2	0.0029(0.0019, 0.0041)	0.0026(0.0018, 0.0035)	<0.001**
C20:3	0.0072(0.0000, 0.015)	0.00001(0.0000, 0.0046)	<0.001**
C20:4	0.0053(0.003, 0.0078)	0.0058(0.0041, 0.0076)	<0.001**
C20:5	0.017(0.0000, 0.0326)	0.0000 (0.0000, 0.0116)	<0.001**
C22:4	0.0003(0.0002, 0.0005)	0.0004(0.0003, 0.0006)	<0.001**

T1, the lowest tertiles of dietary patterns; T3, the highest tertiles of dietary patterns. SFA, saturated fatty acid; MUFA, monounsaturated fatty acid; PUFA, polyunsaturated fatty acid. $^{*}p < 0.05$, $^{**}p<0.01$.

Table S3 Factor-loading matrix for the FAPs

	FAP1	FAP2	FAP3	FAP4	FAP5	FAP6
Cer(18:1/22:0)	0.970*	0.086	0.099	0.102	0.073	-0.031
Cer(18:0/22:0)	0.956*	0.036	0.133	0.082	0.054	-0.031
Cer(18:1/20:0)	0.902*	0.001	0.252	0.036	0.122	0.023
Cer(18:0/24:0)	0.892*	0.140	0.311	0.084	-0.077	0.039
Cer(18:1/24:0)	0.878*	0.204	0.211	0.177	-0.100	0.066
SM(22:0)	0.764*	0.323	0.002	-0.135	0.398	0.002
SM(24:0)	0.704*	0.573	-0.091	-0.05	0.239	-0.038
SM(26:1)	0.021	0.884*	0.16	0.165	0.015	0.095
SM(26:0)	0.270	0.817*	-0.021	0.019	0.037	0.137
SM(24:1)	0.390	0.736*	0.135	0.075	0.185	-0.127
Cer(18:1/18:0)	0.387	0.043	0.802*	0.168	-0.005	0.206
Cer(18:0/18:0)	0.326	0.017	0.756*	0.003	-0.130	-0.120
SM(18:0)	-0.063	0.516	0.721*	-0.134	0.149	0.105
Cer(18:1/24:1)	0.105	0.093	0.110	0.955*	0.064	-0.072
Cer(18:1/22:1)	0.129	-0.031	0.161	0.909*	0.090	-0.035
Cer(18:1/26:1)	-0.044	0.115	-0.111	0.889*	0.040	0.071
SM(14:0)	-0.125	0.312	0.239	-0.236	-0.056	0.748*
Cer(18:1/14:0)	0.177	-0.095	-0.050	0.122	0.332	0.705*
SM(22:1)	0.666	0.382	0.053	-0.231	0.366	-0.058
Cer(18:0/26:0)	0.609	0.407	0.297	0.224	-0.283	0.153
SM(20:0)	0.506	0.335	0.353	-0.282	0.501	0.181
SM(16:0)	0.299	0.518	0.320	0.161	0.302	0.029
SM(20:1)	0.360	0.461	0.367	-0.222	0.364	-0.401
SM(18:1)	0.019	0.503	0.683	-0.092	0.051	-0.248
Cer(18:1/16:0)	0.304	0.034	0.677	0.242	0.438	0.156
Cer(18:0/16:0)	0.134	-0.020	0.621	0.145	0.473	0.154
Cer(18:1/16:1)	-0.013	0.218	0.081	0.262	0.553	0.127
Percentage of variance explained	27.0%	15.2%	14.4%	11.7%	6.9%	5.7%

Cer, ceramide; SM, sphingomyelin; FAP, fatty acid pattern. * Means factor loading with absolute value ≥ 0.7.

Table S4 Demographic characteristics of subjects for lipid analysis

	Total	Control	MCI	*p*
N	50	20	30	
Age	70.0(67.0,75.0)	70.0±4.0	71.8±4.6	0.155
Female, n (%)	25(50.0%)	10(50.0%)	15(50.0%)	0.613
BMR, kcal	1181.0(1112.0,1393.5)	1298.5(1137.3,1467.0)	1129.5(1090.3,1342.3)	0.019*
Education, n (%)				0.840
Illiterate	12(24.0%)	5(25.0%)	7(23.3%)	
Primary school	18(36.0%)	8(40.0%)	10(33.3%)	
Junior high school	16(32.0%)	5(25.0%)	11(36.7%)	
High school and above	4(8.0%)	2(10.0%)	2(6.7%)	

BMR, basal metabolic rate, MCI, mild cognitive impairment. $^{*}p < 0.05$.
